# Myeloma-derived macrophage inhibitory factor regulates bone marrow stromal cell-derived IL-6 via c-MYC

**DOI:** 10.1186/s13045-018-0614-4

**Published:** 2018-05-16

**Authors:** Rachel E. Piddock, Christopher R. Marlein, Amina Abdul-Aziz, Manar S. Shafat, Martin J. Auger, Kristian M. Bowles, Stuart A. Rushworth

**Affiliations:** 1 Department of Molecular Haematology, Norwich Medical School, The University of East Anglia, Norwich Research Park, Norwich, NR4 7TJ UK; 2 Department of Haematology, Norfolk and Norwich University Hospitals NHS Trust, Colney Lane, Norwich, NR4 7UY UK

**Keywords:** Myeloma, MIF, cMYC, BMSC, Stromal, IL-6, IL-8, Bone marrow

## Abstract

**Abstract:**

Multiple myeloma (MM) remains an incurable malignancy despite the recent advancements in its treatment. The protective effects of the niche in which it develops has been well documented; however, little has been done to investigate the MM cell’s ability to ‘re-program’ cells within its environment to benefit disease progression. Here, we show that MM-derived macrophage migratory inhibitory factor (MIF) stimulates bone marrow stromal cells to produce the disease critical cytokines IL-6 and IL-8, prior to any cell-cell contact. Furthermore, we provide evidence that this IL-6/8 production is mediated by the transcription factor cMYC. Pharmacological inhibition of cMYC in vivo using JQ1 led to significantly decreased levels of serum IL-6—a highly positive prognostic marker in MM patients.

**Conclusions:**

Our presented findings show that MM-derived MIF causes BMSC secretion of IL-6 and IL-8 via BMSC cMYC. Furthermore, we show that the cMYC inhibitor JQ1 can reduce BMSC secreted IL-6 in vivo, irrespective of tumor burden. These data provide evidence for the clinical evaluation of both MIF and cMYC inhibitors in the treatment of MM.

**Electronic supplementary material:**

The online version of this article (10.1186/s13045-018-0614-4) contains supplementary material, which is available to authorized users.

Despite significant recent advancements made in the treatment of multiple myeloma (MM), relapse remains inevitable and the disease presently remains incurable. This is attributable, in part, to the highly protective nature of the BM micro-environment niche in which the malignant plasma cells proliferate. Macrophage migratory inhibitory factor (MIF) is a cytokine associated with various roles [1] and is rapidly developing a pro-tumoral identity [2]. Elevated MIF levels are described in MM and have been implicated in MM bone marrow homing and chemotherapy resistance [3]; however, the adaptive effect that MM-derived MIF has on the tumor microenvironment is not yet defined. Here, we investigate the function of MM-derived MIF in the MM microenvironment by examining its effects on bone marrow stromal cells (BMSC).

We and others have found that MM cells have significantly elevated MIF gene expression and secreted protein levels [3] (Additional file 1: Figure S1). MIF KD resulted in lower MM proliferation in BMSC co-culture (Additional file 1: Figure S2C) alongside reduced tumor burden and improved overall survival in vivo (Fig. 1a, b). To investigate the effects of MIF secretion by MM on its microenvironment, we used cytokine arrays to establish if cytokine changes occur when MM cells are cultured with primary BMSC. Elevated levels of IL-6/8 were detected in co-culture experiments when compared with either BMSC or MM monoculture arrays (Fig. 1c, d); no MIF was detected in BMSC cultured alone. Cytokine array analysis of the supernatant from MIF-stimulated BMSCs confirmed this IL-6/IL-8 secretion (Fig. 1e, f) and was quantified via ELISA (Additional file 2) in Fig. 1g. Pre-treatment of BMSC with the MIF inhibitor ISO-1 significantly reduced the MIF induction of IL-6/IL-8 by BMSC (Fig. 1h). BMSC expressed all three known receptors for MIF (CXCR4, CXCR2 and CD74); however, only blocking CD74 inhibited MIF-Induced IL6/8 (Additional file 1: Figure S3A&B).

**Fig. 1:**
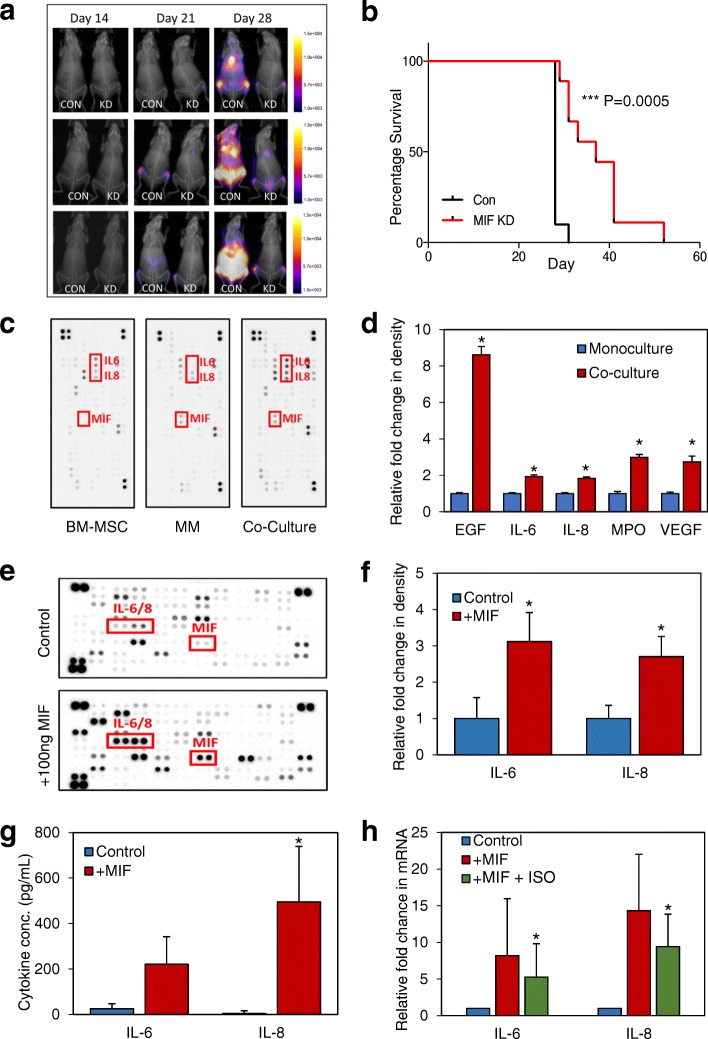
MM derived MIF is pro-tumoral and drives BMSC IL-6 and IL-8. 1 × 10^6^ MM.1S-luc cells (ShE control *n* = 10, and ShMIF *n* = 7) were injected via the tail vein of 6–8-week-old NSG mice. **a** Mice were monitored weekly by bioluminescent imaging. **b** Kaplan-Meier curve showing survival, analyzed using Mantel Cox regression. **c** Representative (*n* = 3) Human XL cytokine array output after a 24-h incubation in either mono or co-culture, cell supernatant was used for analysis. **d** Graphical representation of **c**—values for BMSC and MM monoculture intensities were added together and were analyzed against co-culture experiment signal intensity using HL++ image software which show differences in several key cytokines. **e**, **f** BMSC were stimulated with 100 ng/mL of human recombinant MIF and incubated for 24 h; supernatant was used for assay. Representative (*n* = 3) image of cytokine array (**e**) and subsequent graphical representation (**f**) of analysis using HL++ software. **g** Primary BMSC (*n* = 4) were stimulated with 100 ng/mL recombinant human MIF for 6 h after which IL-6 and IL-8 protein excretion was analyzed by ELISA. **h** Primary BMSC (*n* = 4) were incubated with/without 10μg ISO-1 and then stimulated with 100 ng/mL recombinant human MIF for 6 h. IL-6 and IL-8 transcriptional levels were then analyzed by RT-PCR

An inhibitor panel was used to screen for potential pathways responsible for MIF-induced BMSC-derived IL-6/IL-8 expression, and we found that JQ1 inhibited MIF induced IL-6 and IL-8 (Fig. 2a, b) mRNA in BMSC. We then determined if JQ1 could regulate BMSC pro-tumoral interleukin production in-vivo. Although BMSC are often cyto-protective in the context of anti-MM therapy, others have observed that the sensitivity of MM cell lines to JQ1 was unchanged by the presence of HS-5 [4]. Following MM engraftment (Fig. 2d), mice were randomized and treated for 5 days with I.P injections of 50 mg/kg JQ1 or alternatively vehicle control. Under 7 days of treatment with JQ1 was predicted to have no measurable effect on tumor burden [4] and was selected to control for the effects of tumor burden on MM-MIF secretion and subsequent BMSC IL-6 expression. We found no difference in MM burden between groups (Fig. 2d, f). Nevertheless, murine IL-6 was significantly reduced in the JQ1-treated animals (carrying human MM) compared to control animals (Fig. 2e).

**Fig. 2:**
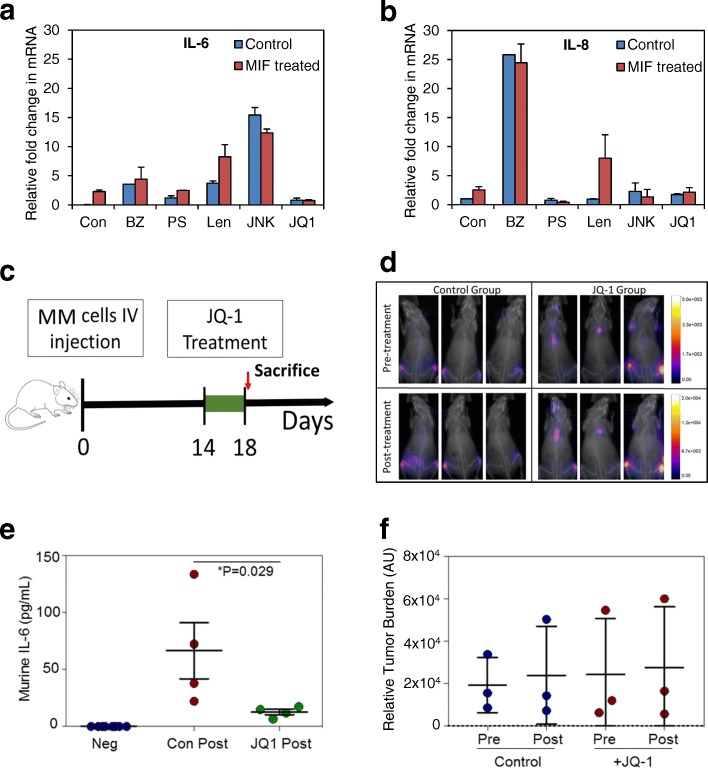
MM-derived MIF regulates bone marrow stromal cell-derived IL-6 and IL-8 via cMYC. **a**, **b** BMSC cells were pretreated with various drugs (bortezomib 10 nM, PS341 100 nM, Lenolidiomide 500 nM, JNKV 10uM, and JQ1 500 nM) for 30 mins and then activated with MIF for 2 h. RNA was extracted, and transcriptional levels of IL-6 (**a**) or IL-8 (**b**) was analyzed. **c** Schematic showing JQ1 in vivo experiment. 1 × 10^6^ U266 cells were injected via the tail vein of NGS mice (*n* = 8). Following a 2-week engraftment period, mice were treated with 50 mg/kg JQ1 or vehicle control daily for 5 days, after which all mice were sacrificed. **d** Mice were monitored pre and post treatment with JQ1 by bioluminescent imaging which was quantified (**f**) by ImageJ densitometry. **e** ELISA data showing murine IL-6 serum concentration following JQ1 treatment. Baseline levels of murine IL-6 were non-detectable in mice without MM

Despite MIF’s association with hematological malignancies, previous work has focused on the effects of MIF on the malignant cells rather than the supportive cells of the microenvironment [3]. Here, we show that MM-derived MIF is pro-tumoral through induction of BMSC-derived IL-6/8. IL-6 is central to MM pathogenesis and primarily comes from the BMSC in the tumor micro-environment [5]. Here, we place IL-6 downstream of MIF-induced BMSC activation [6, 7]. IL-8 expression in BMSC, which has been shown to parallel MM disease progression [8] and positively influence osteoclastogenesis in MM [9], was increased in BMSC in response to MIF. Furthermore, the BET-bromodomain inhibitor JQ1 significantly decreased IL-6/8 secretion in MIF-stimulated BMSC. In vivo use of JQ1 significantly reduced levels of murine IL-6 in the serum [5, 10, 11]. Taken together, this suggests that JQ1 is exerting anti-MM activity, in part, through a direct effect on BMSC via the inhibition of BMSC IL-6 (and IL-8) synthesis. This in turn could explain why BMSC do not appear to offer MM protection from JQ1 therapy.

## Additional files

Additional file 1: **Figure S1.** (A) Relative transcriptional levels of MIF expression in B cells, T cells, non-malignant plasma cells and CD138+ purified primary MM cells were normalized to Beta-Actin (*n* = 5). (B) MIF ELISA data showing the increase in MIF extracellular protein levels in CD138+ purified primary MM cells and MM cell lines in comparison to other cell types (*n* = 5). **Figure S2.** (A) MM.1 s cells were transduced with lentivirus targeted to MIF or control shRNA for 96 h. RNA was extracted and MIF mRNA expression was analyzed via RT-PCR to confirm KD. (B) Cells described in (A) were cultured for 24 h in fresh media after which the supernatant was analyzed for MIF via ELISA. (C) MM.1S MIF KD cells were co-cultured with primary BMSC for 96 h, after which MM cells were analyzed using Cell Titre Glo (CTG), normalized to a ShE control. **Figure S3**. (A) BMSC from MM patients was analyzed for CXCR4, CXCR2 and CD74 using flow cytometry and compared to isotype control. (B) BMSC were preincubated with either AMD3100 (10 μM), SB225002 (100 nM) and anti-CD74 (10 μg/ml). BMSC were then stimulated with recombinant MIF (100 ng/ml) for 2 h. RNA was extracted and analyzed for IL-6 and IL-8 expression using Real-time PCR. (PPTX 178 kb)

Additional file 2: Supplementary methods. Methods section. (DOCX 19 kb)

## Abbreviations

BMSC: Bone marrow stromal cell
IL: Interleukin
MIF: Macrophage migratory inhibitory factor
MM: Multiple myeloma
NSG: NOD.Cg-Prkdcscid IL2rgtm1Wjl/SzJ
