# Supplementary figures and images for: Myeloma-derived macrophage inhibitory factor regulates bone marrow stromal cell-derived IL-6 via c-MYC
Source: J Hematol Oncol. 2018 May 16;11:66. doi: 10.1186/s13045-018-0614-4 (PMC5956761; doi:10.1186/s13045-018-0614-4)

## Slide 1
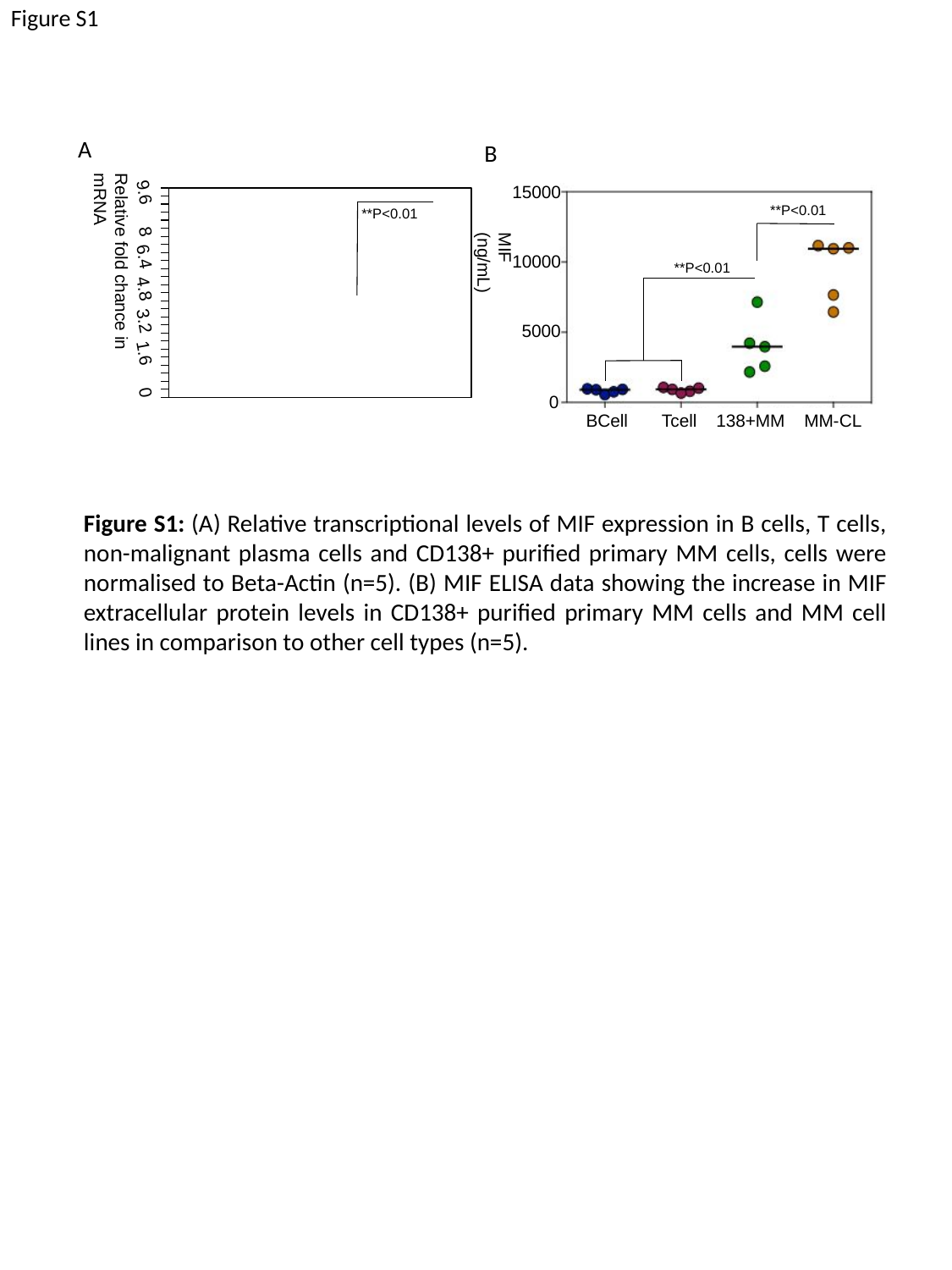

## Slide 2
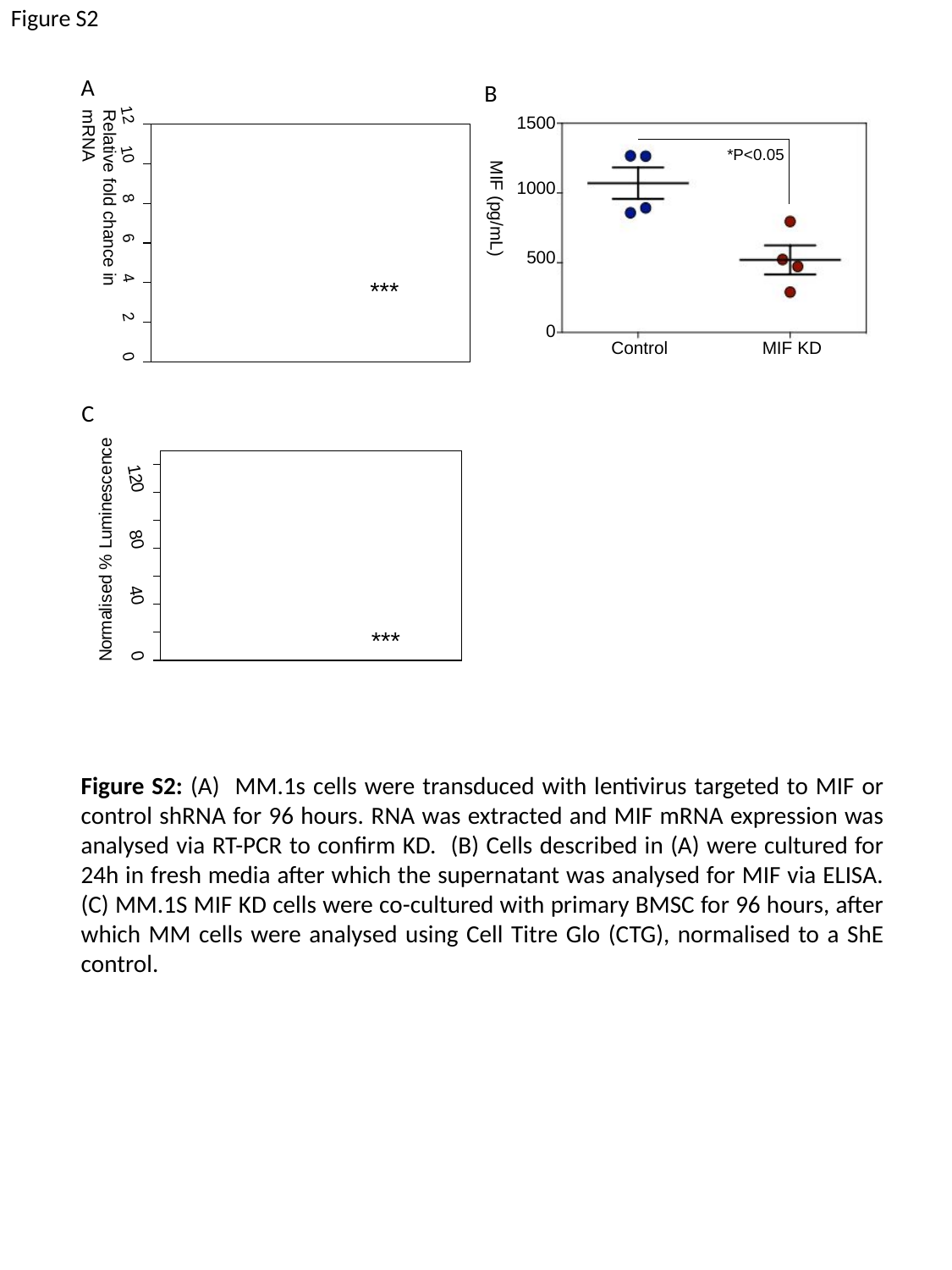

## Slide 3
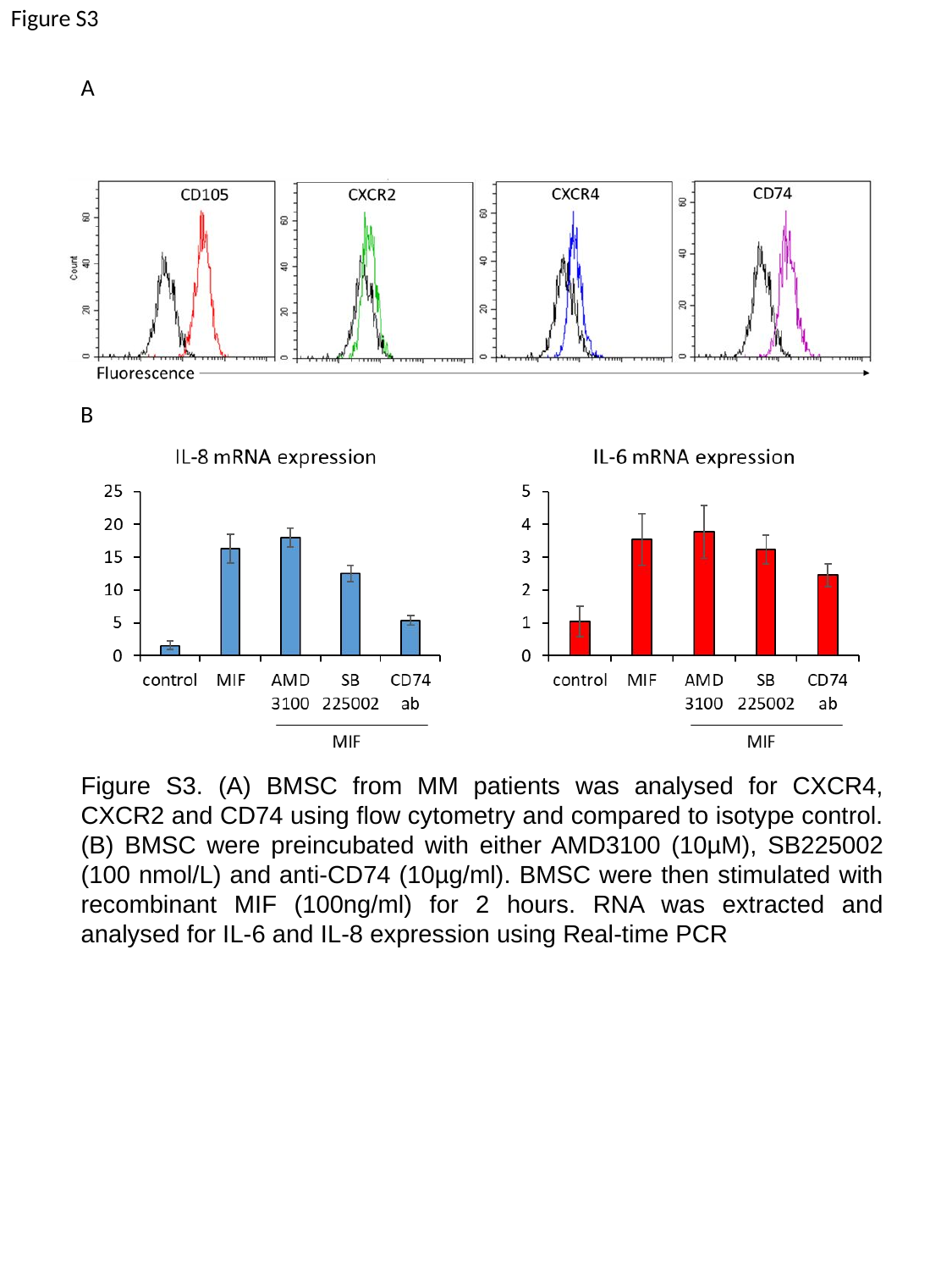

Supplement: Supplementary file 1 — Figure S1. (A) Relative transcriptional levels of MIF expression in B cells, T cells, non-malignant plasma cells and CD138+ purified primary MM cells were normalized to Beta-Actin (n = 5). (B) MIF ELISA data showing the increase in MIF extracellular protein levels in CD138+ purified primary MM cells and MM cell lines in comparison to other cell types (n = 5). Figure S2. (A) MM.1 s cells were transduced with lentivirus targeted to MIF or control shRNA for 96 h. RNA was extracted and MIF mRNA expression was analyzed via RT-PCR to confirm KD. (B) Cells described in (A) were cultured for 24 h in fresh media after which the supernatant was analyzed for MIF via ELISA. (C) MM.1S MIF KD cells were co-cultured with primary BMSC for 96 h, after which MM cells were analyzed using Cell Titre Glo (CTG), normalized to a ShE control. Figure S3. (A) BMSC from MM patients was analyzed for CXCR4, CXCR2 and CD74 using flow cytometry and compared to isotype control. (B) BMSC were preincubated with either AMD3100 (10 μM), SB225002 (100 nM) and anti-CD74 (10 μg/ml). BMSC were then stimulated with recombinant MIF (100 ng/ml) for 2 h. RNA was extracted and analyzed for IL-6 and IL-8 expression using Real-time PCR. (PPTX 178 kb) [file 13045_2018_614_MOESM1_ESM.pptx]
