# Supplementary material for: Myeloma-derived macrophage inhibitory factor regulates bone marrow stromal cell-derived IL-6 via c-MYC
Source: J Hematol Oncol. 2018 May 16;11:66. doi: 10.1186/s13045-018-0614-4 (PMC5956761; doi:10.1186/s13045-018-0614-4)
Supplement: Supplementary file 2 — Supplementary methods. Methods section. (DOCX 19 kb) [file 13045_2018_614_MOESM2_ESM.docx]

**Supplementary**

**Materials and Methods:**

**Materials**

Recombinant MIF, Proteome Profiler Human XL arrays and IL-6/8 ELISAs were purchased from R&D systems (Oxford, UK). MIF ELISA was obtained from BioLegend (London, UK). Inhibitors SP600125 (JNK), lenolidomide, bortezomib, PS1145, JQ1, SB225002, and AMD3100 were purchased from Selleck Chemicals (Cambridge, UK). Anti-CD74 blocking antibody was purchased from BD Biosciences. Flow cytometry antibodies against CXCR4, CXCR2 and CD74 were obtained from Miltenyi Biotech (Bergisch Gladbach, Germany). Other antibodies were obtained from Cell Signaling Technology (Cambridge, MA, USA), with the exception of anti-cMyc, which was purchased from Abcam, UK. All other reagents were purchased from Sigma-Aldrich (St Louis, MO, USA).

**Cell lines and Primary Samples**

The MM-derived cell lines were obtained from the European Collection of Cell Cultures where they are authenticated by DNA-fingerprinting. Myeloma cell lines were cultured in RPMI 1640 medium supplemented with 10% fetal bovine serum (FBS), and 1% penicillin/streptomycin (all obtained from Invitrogen, Paisley, UK). Primary MM cells were obtained from patients’ bone marrow after informed consent was given in accordance with the Declaration of Helsinki and under approval from the Health Research Authority of the National Health Service, United Kingdom (07/H0310/146).

For primary cell isolation, heparinized blood was collected from volunteers and human peripheral blood mononuclear cells were isolated by histopaque density-gradient centrifugation and plated in growth media. Non-adherent cells were removed after 24 hours and primary plasma cells were purified by positive selection using magnetic-activated cell sorting with CD138^+^ MicroBeads (Miltenyi Biotec, Auburn, CA). At approximately 80% confluency, adherent cells were trypsinized and expanded in Dulbecco’s Modified Eagle’s Medium (DMEM) with 20% FBS and 1% penicillin/streptomycin for 3 to 5 weeks.

**Real-time PCR**

Total RNA was extracted from cells using Promega ReliaPrep kit as per the manufacturer’s instructions. Reverse transcription was performed using an RNA polymerase chain reaction (PCR) core-kit (Applied Biosystems). Relative quantitative real-time (qRT)-PCR used SYBR-green technology (Roche) on generated complementary DNA. After pre-amplification (95°C/120 seconds), PCR products were amplified for 45 cycles (95°C/15 seconds, 60°C/10 seconds, 72°C/10 seconds) on a Roche 384-well LightCycler480. Messenger RNA (mRNA) expression was normalized against B-actin.

**Cytokine Arrays and ELISAs**

Cytokines were analyzed using the Proteome Profiler™ Human XL Cytokine Array Kit (R&D systems - Minneapolis, MN). Briefly, MM cells (0.5x10^6^) and confluent BMSC were incubated for 24 hours in mono- or co-culture. Supernatants were then collected for analyses using the array kit as specified by the manufacturer. Mean intensities of samples on the membrane, representative of relative protein levels, were then quantified using HL++ image software. ELISAs were performed as per the manufacturer’s instructions.

**Lentiviral transduction**

pCDH-luciferase-T2A-mCherry was kindly donated from Professor Irmela Jeremias, Helmholtz Zentrum München, München, Germany [1], MIF Mission shRNA Glycerol stock (TRCN0000056818) was purchased from Sigma Aldrich and lentiviral particles using these constructs were generated as previously described [2]. Lentiviral stocks were concentrated and quantified using Amicon Ultra centrifugal filters and Lenti-X qRT-PCR titration kit (Clonetech, Oxford, UK) respectively. Transductions were performed on cell lines as previously described [3]. MM.1S cells expressing mCherry (MM.1S-luc) were sorted on a FACSAria (BD, Oxford, UK) prior to MIF further knockdowns. MIF knockdown was confirmed using qRT-PCR.

**Flow Cytometry**

CyFlowCube 6 (Sysmex, Milton Keynes,UK) was used for flow cytometry analysis. Cells were incubated for 5 minutes with the FCR receptor blocker (Miltenyi Biotec; Catalog number 130-059-901) and then stained with isotype controls or test antibodies (Miltenyi Biotec). Gates were set to the appropriate isotype control.

**MM xenograft model**

NOD.Cg-Prkdcscid IL2rgtm1Wjl/SzJ (NSG) mice from The Jackson Laboratory (Bar Harbour, ME, USA) were maintained under specific pathogen-free conditions in the research animal facility of The Disease Modelling Unit, The University of East Anglia, Norwich, UK. All animal experiments were performed in accordance with UK Home Office and University of East Anglia Animal Welfare Ethics Review Board regulations. MIF knockdown xenograft model: 0.5x10^6^ MM.1S-luc cells (ShE = 10, ShMIF = 7) were intravenously (IV) injected into non-irradiated 6-8 week old NSG mice on Day 1 of experiment. c-Myc inhibition via JQ1: non-irradiated 6-8 week old NSG mice (n=8) were injected with 0.5x10^6^ U266-luc cells via tail vein. After a two week engraftment period, mice were treated with JQ1 at 50mg/kg/day (n=5) or a vehicle control (n=4). PB samples were taken via tail vein at day 13 and day 18 (pre and post treatment respectively). Upon clinical signs of illness, mice were sacrificed via CO_2_ exposure. Mice were monitored via *in vivo* bioluminescent imaging (Bruker, Coventry, UK) throughout.

**Statistical Analysis**

Statistical analyses were performed using Student’s T test or Mann-Whitney U test unless otherwise stated. The Mantel-Cox test was used to analyze Kaplan-Meier survival data. Results with P<0.05, P<0.01 and P<0.001 are denoted ‘*’, ‘**’ and ‘***’ respectively and are considered statistically significant. Statistical analysis was performed using Graphpad Prism 5 software (Graphpad, San Diego, CA, USA)

1. Vick B, Rothenberg M, Sandhöfer N, Carlet M, Finkenzeller C, Krupka C, et al. An advanced preclinical mouse model for acute myeloid leukemia using patients' cells of various genetic subgroups and in vivo bioluminescence imaging. PloS one. 2015;10(3):e0120925

2. Rushworth SA, Zaitseva L, Murray MY, Shah NM, Bowles KM, MacEwan DJ. The high Nrf2 expression in human acute myeloid leukemia is driven by NF-κB and underlies its chemo-resistance. Blood. 2012;120(26):5188-98

3. Pillinger G, Loughran NV, Piddock RE, Shafat MS, Zaitseva L, Abdul-Aziz A, et al. Targeting PI3Kδ and PI3Kγ signalling disrupts human AML survival and bone marrow stromal cell mediated protection. Oncotarget. 2016;7(26):39784-95
